# Efficacy of mHealth and education-led peer counseling for patients with hypertension and coronary artery disease in Pakistan: study protocol for a double-blinded pragmatic randomized-controlled trial with factorial design

**DOI:** 10.1186/s13063-023-07472-0

**Published:** 2023-07-10

**Authors:** Muhammad Arshed, Rubeena Zakar, Muhammad Farooq Umer, Mehwish Kiran, Najeeb Ullah, Ghazala Iftikhar, Florian Fischer

**Affiliations:** 1 Department of Community Health, Faculty of Medicine and Health Sciences, Universiti Putra Malaysia, Serdang, Malaysia; 2 Department of Public Health, Institute of Social and Cultural Studies, University of Punjab, Lahore, Pakistan; 3 Preventive Dentistry Department, College of Dentistry, King Faisal University, Hofuf, Saudi Arabia; 4 Department of Gynaecology and Obstetrics, Punjab Employees Social Security Institute, Lahore, Pakistan; 5 Department of Cardiology, Rehmatul lil Alameen Institute of Cardiology, Lahore, Pakistan; 6 Institute of Public Health, Charité – Universitätsmedizin Berlin, Berlin, Germany; 7 Bavarian Research Center for Digital Health and Social Care, Kempten University of Applied Sciences, Kempten, Germany

**Keywords:** Mobile health, eHealth, Intervention, Medication adherence, Hypertension, Systolic blood pressure

## Abstract

**Background:**

Hypertension is a highly relevant public health challenge. Digital interventions may support improving adherence to anti-hypertensive medications and alter health behavior. Therefore, this protocol describes a study that aims to assess the effectiveness of mHealth and educational support through peer counseling (Ed-counselling) to control blood pressure in hypertensive patients when compared to standard care.

**Methods:**

We chose a double-blinded pragmatic randomized-controlled with factorial design for this investigation. The trial is going to recruit 1648 hypertensive patients with coronary artery disease at the age of 21 to 70 years. All participants will already be on anti-hypertensive medication and own a smartphone. They will be randomized into four groups with each having 412 participants. The first group will only receive standard care; while the second group, in addition to standard care, will receive monthly Ed-counselling (educational booklets with animated infographics and peer counseling); the third group will receive daily written and voice reminders and an education-led video once weekly together with standard care; while the fourth one gets both interventions given to second and third groups respectively. All groups will be followed-up for 1 year (0, 6, and 12 months). The primary outcome will be the change in systolic blood pressure while secondary outcomes include health-related quality of life and changes in medication adherence. For measuring changes in systolic blood pressure (SBP) and adherence scores difference at 0, 6, and 12 months between and within the group, parametric (ANOVA/repeated measure ANOVA) and non-parametric tests *(*Kruskal*-*Wallis test*/*Friedman test*)* will be used. By using the general estimating equation (GEE) with negative binomial regression, at 12 months, the covariates affecting primary and secondary outcomes will be determined and controlled. The analysis will be intention-to-treat. All the outcomes will be analyzed at 0, 6, and 12 months; however, the final analysis will be at 12 months from baseline.

**Discussion:**

Besides adding up to existing evidence in the literature on the subject, our designed modules using mHealth technology can help in reducing hypertension-related morbidity and mortality in developing countries.

**Supplementary Information:**

The online version contains supplementary material available at 10.1186/s13063-023-07472-0.

## Background

Hypertension among adults is an increasing public health challenge that affects 1.13 billion people around the globe [1] and is considered a major cause of mortality resulting in 9.4 million deaths worldwide per year [2]. About 75% of all hypertension cases are diagnosed in low-and middle-income countries (LMICs) [1, 3]. The risk of cardiovascular events can be reduced to about one-half for every 20 mmHg decrease in systolic blood pressure and 10 mmHg in diastolic blood pressure [4]. Likewise, cardiovascular disease-related deaths are preventable and can be better managed by reducing systolic blood pressure [5].

In Pakistan, 18.9% of teenagers over the age of 15 years and 33% of adults over the age of 45 years suffer from hypertension [6]. Adherence is termed as “the extent to which individuals follow their healthcare providers’ prescribed drug schedules” [7]. Adherence to cardiac medications was reported at 77% [8], while non-adherence to antihypertensive medication was found among 37.7% of patients in Pakistan [9]. Thus, non-adherent patients constitute a significant proportion of the treatment population in the country. Also, the majority of individuals on antihypertensive medications had uncontrolled blood pressure and poor adherence to treatment which further compounds the dysregulation of an individual’s blood pressure [9, 10].mHealth is a term used for any medical and public health application supported by mobile phones, personal digital assistants, patient monitoring devices, or other wireless devices [11]. The use of mobile health interventions to enhance medication adherence has been reported to be effective, particularly in LMICs [12–14] as these provide results at a lowered cost [15]. Despite positive feedback reported on the effectiveness of mHealth interventions on adherence to medication in general, its role in cardiovascular medication adherence is under-studied especially in a scarcely resourced country like Pakistan. Moreover, in the context of behavior changes in response to the changing dynamics of disease, there is a need to compare mHealth intervention with different interventional methods to assess its effectiveness [16]. Similarly, the literature suggests that counseling therapy is also an effective strategy in modifying behavior regarding adherence to antiretroviral and anti-tuberculosis treatment [17–19]. Counseling was found to be an affordable intervention at the personal level in enhancing medication adherence [20] but its use in hypertension and cardiovascular diseases is limited.

There is a need for an efficient and cost-effective model to be adopted in LMICs like Pakistan, where there is a lack of resources (e.g., constraints on the public health budget) and where people have a generally poor educational status. Most of the interventions in improving medication adherence are not financially affordable besides having complex designs. It is therefore imperative to work on improving adherence to anti-hypertensive medications and altering behavior by designing cost-effective interventions with lesser intricacies.

This study is based on the experiences of a previous study that has been conducted as a doctoral thesis by the first author. We are going to investigate the effectiveness of two modules that are designed for this study. The first module is Ed-counselling and the second one is a mHealth intervention in altering beliefs regarding adherence to treatment and lowering systolic blood pressure in hypertensive patients with coronary artery diseases. This research will compare these two models to know which one is more accepted and effective in the Pakistani population. This study is expected to yield substantial findings and may aid in the development of educational guidelines for patients to increase drug adherence and reduce untoward consequences.

### Research objectives

- To determine the mean differences in treatment outcome (systolic blood pressure) among the four groups at 0, 6, and 12 months after the intervention
- To assess the effect of newly developed intervention modules on health beliefs related to treatment outcome (systolic blood pressure change) among patients with hypertension on anti-hypertensive treatment
- To determine the mean differences in (medication) adherence among the four groups at 0, 6, and 12 months after the intervention
- To measure the health-related quality of life at 0, 6, and 12 months after the intervention

## Methods

### Study design

**Fig. 1:**
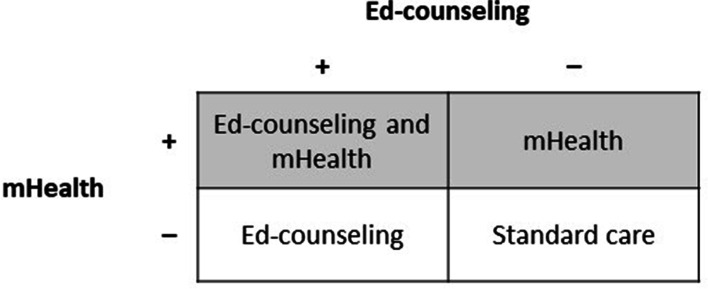
Factorial distribution of the groups in the trial: mHealth, Ed-counseling, and standard care

It is a factorial randomized controlled trial with a superiority design and a double-blinding approach. Intention-to-treat analysis with 12 months of follow-up will be applied to determine the efficacy of Ed-counselling assistance and mHealth intervention on hypertensive patients at four teaching hospitals in Lahore, Pakistan. Lahore is the provincial capital of Punjab (the largest province in the country) [21]. It is the world’s eighteenth-largest city and Pakistan’s second-largest metropolis. The SPIRIT 2013 Statement (Standard Protocol Items: Recommendations for Interventional Trials) [22] provides the basis for this research. A factorial approach will be used to assign participants to the four groups. The groups will receive standard care, Ed-counselling, mHealth intervention, and combined intervention (Fig. 1). All groups will be followed up for a period of total 12 months. Systolic blood pressure, the Self-efficacy for Appropriate Medication Adherence Scale (SEAMS), the number of prescribed tablets taken for a specified period (self-reporting), and health-related quality of life will be measured at least three times: at baseline, 6 months, and 12 months after the intervention [23, 24].

### Study participants and eligibility criteria

The study participants will be hypertensive patients with coronary artery disease as comorbidity registered in the Cardiology Outpatient Departments (OPDs) of four selected public teaching hospitals in Lahore, Pakistan.

Inclusion criteria include men and women over the age of 21 and up to 70 years, who have been registered for the last 30 days or more as hypertensive with comorbidity of coronary artery disease in the OPDs of the selected teaching hospitals in Lahore, Pakistan. These participants will be having stable coronary artery disease, are already on anti-hypertensive medication, are willing to sign a written informed consent form, possess a smartphone with access to the Internet, and are able to read Urdu/English.

Patients with a history of cancer who may need drug changes; with a blood pressure reading of more than 220/120 mmHg; with any biological condition that makes it difficult for them to read, write, communicate, or hear phone calls; with pregnancy and in lactation period; and who are enrolled in some other study will be excluded.

### Sample size

For comparing different interventions among the four groups, the sample size is computed through the STATA software using a standard deviation of 18.5 in systolic blood pressure taken from a Pakistani study [9], population means of 148 and 143 respectively, as 5 mmHg reduction in systolic blood pressure is related to clinical importance regarding decreasing the risk of coronary artery disease [25]. A two-sided level of significance of 5% and a power of 90% with a 30% attrition rate between the baseline and 12 months of the intervention period were used. The total sample size calculated is 1648 with 412 in each group.

### Recruitment

After the screening, the participants fulfilling the eligibility criteria will be recruited. For this purpose, a trained doctor in the department of cardiology from each teaching hospital in the study will facilitate and monitor the recruitment process. Each of these doctors will be supported by one research assistant who will act as the project’s focal person in each hospital for the entire study duration. The contact information of participants who have been recruited will be gathered and saved.

After shortlisting, participants will be briefed (written and verbally) about the study and presented with consent forms bearing the option to relinquish their participation from the study at any moment of its duration. Participants will subsequently be enrolled and assigned code numbers by the principal investigator. All these participants will be able to select their communication language of choice (Urdu and/or English), as well as the day and hour of their respective sessions.

### Randomization and blinding

To avoid any potential bias, a simple complete randomization procedure will be utilized [26]. A computer-generated, simple complete random approach will be used to divide participants into four groups [27]. To keep their identities hidden, all participants will be assigned an identification number. To keep the allocation of participants into four groups hidden from the research staff, tight concealment of allocation will be observed. Before allotment, written assignments will be sealed in opaque packets with tagged identifying numbers [28].

All subsequent randomization processes will be completed by an independent biostatistician who will no longer be a participant in the trial. Independent personnel will be involved in the randomization, evaluation, and intervention delivery. The staff involved in data collection and outcome assessment will be blinded. They will be unaware of the allocation of any group [29].

**Fig. 2:**
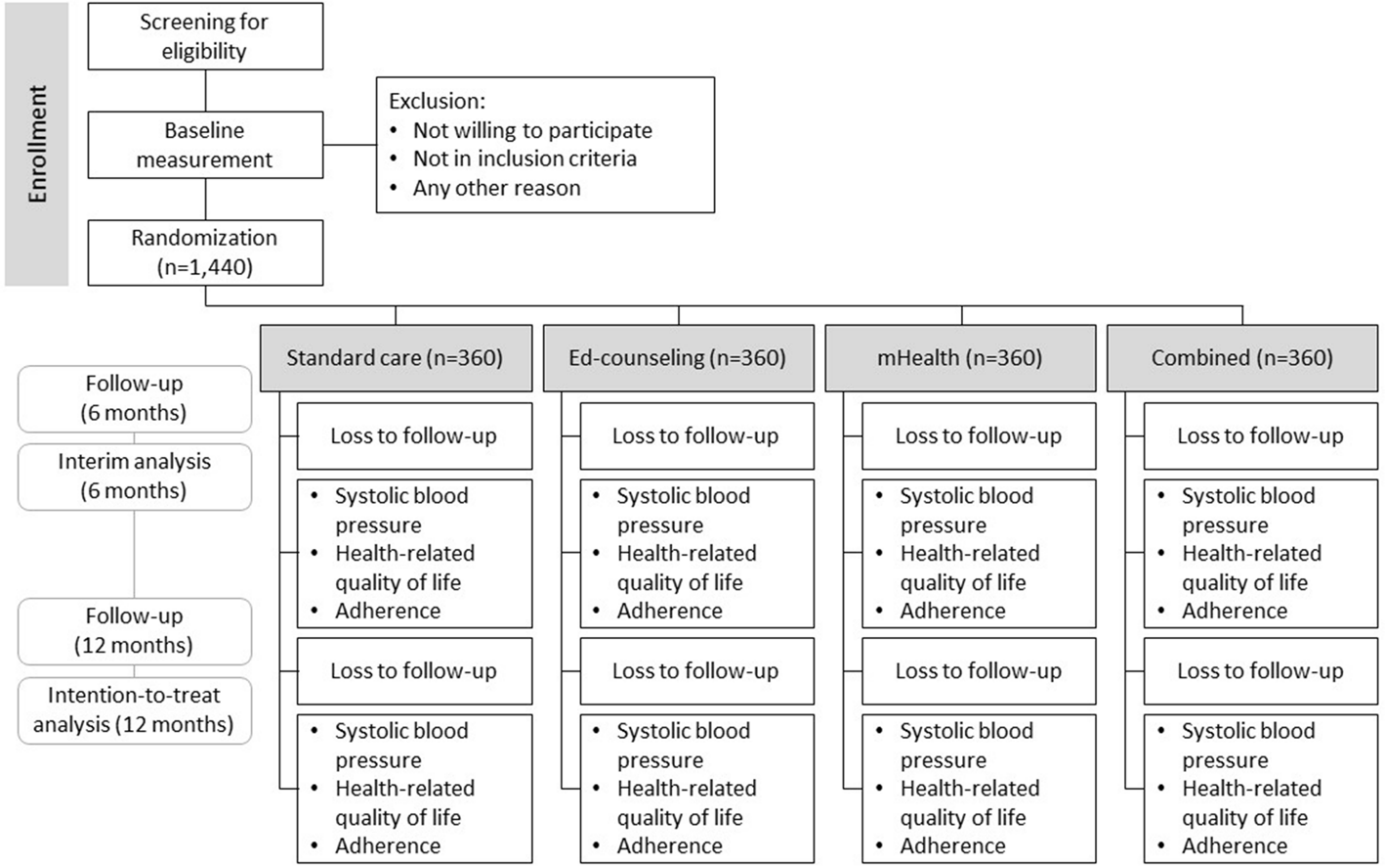
Flow diagram

The recruitment process will continue until a final sample size of 1648 people from four teaching hospitals is reached. Participants’ follow-up period is 12 months from the point of randomization (Fig. 2).

### Unblinding

For our study, unblinding is not of crucial importance as this study does not execute a drug trial. Yet, we shall take care of the unblinding procedure. Unblinding at any stage of the trial shall be done by an independent statistician not involved in the main trial so that other blinded procedures may not be affected. After an alert is raised by the on-site physician, the principal investigator shall analyze the situation and call for the unblinding. The database containing the results of the nested study and their corresponding data from the main trial of the unblinded participants will be emailed to the statistician who will further process the data.

### Intervention

The Health Belief Model is used to create the content of the study modules (Table 1). Expert consultations in the domains of behavioral sciences, health education, information technology, cardiac medicine, and hypertension management were sought in the development of these modules. The goal of these interventions is to control systolic blood pressure among study participants after 12 months.

**Table 1:** Description of the different arms with intervention

**Arms**	**Components**	**Description**
**Ed-counseling intervention**	**Educational support** (educational booklet with animated infographics)	1) Ensuring that professionals are available throughout the continuum of care and establishing team-based care; 2) educating and empowering patients so that they are aware of the treatment plan and its advantages; 3) lowering the cost of medication, as well as lowering the barriers to accessing it. Education on hypertension, blood pressure self-monitoring, and frequent systolic blood pressure tests, as well as body weight and serum cholesterol impact on systolic blood pressure, are included in the instructional support. Importance of medication adherence. The real need for a healthy lifestyle with a heart-friendly diet.
	**Peer counseling (family member)**	• The counseling sessions are arranged face to face, and a specially developed module to change the beliefs of participants related to treatment outcome and medication adherence • This unique module is developed to change their beliefs related to poor lifestyle • The counseling sessions are geared toward overcoming the general obstacles related to medication adherence and poor lifestyle • The distinct feature of the counseling component centered on personal barriers • The involvement of family support will make it an effective tool • Counseling and encouraging participants to stick with their doctor and medication, even if they feel better. • Changing their behavior towards a healthy lifestyle • Proper medication adherence and adopting a healthy lifestyle eventually help them out in improving health-related quality of life
**mHealth intervention**	**Written and voice reminders**	• “It’s time for you to take your medicine.” • “This is to remind you that you need to take your medication.” • “How are you?” “Remember your appointment a day after tomorrow” • “How are you?” “Remember your appointment tomorrow”
	**Educational-led video**	• Awareness about hypertension • What causes uncontrolled hypertension • Consequences of uncontrollable hypertension • Effect of medication adherence on blood pressure • Effect of healthy lifestyle change on blood pressure • Effect of medication adherence and healthy lifestyle on quality of life • Relation of poor adherence (medication and lifestyle) with uncontrolled hypertension • Lifestyle changes for better health outcome, which enhances better health-related quality of life
**Combined intervention**	**Ed-counseling intervention**	Ed-counseling intervention + mHealth intervention
	**mHealth intervention**	

The first group will receive no intervention and only standard hospital care (as per the practice of the hospital); the second group will receive monthly Ed-counselling sessions in addition to standard care; the third group will receive daily written and voice reminders and once weekly an education-led video in addition to standard care; the fourth group will receive Ed-counselling sessions every month, daily written and voice reminders, and once weekly an education-led video in addition to standard care.

#### Educational support with peer counseling

Peer counseling sessions will be led by doctors who are specialized in hypertension. Patients and their families will be focused on these sessions. Face-to-face sessions will take roughly 20–25 min each. Patients will receive spoken and written instructions based on their abilities during these sessions. Counseling sessions are geared toward overcoming both general and personal obstacles. They will learn about the need of adhering to medicine, nutrition, and exercise regimens, as well as about the possible consequences of failing to do so (heart attack, stroke, nephropathy, retinopathy, and dementia). The counseling component also addresses personal barriers to taking medication and is designed to assist participants in better understanding their drug-taking attitude. The peer counseling sessions will take place four times for a total of twelve months, at 1, 4, 8, and 12 months. Information on hypertension, blood pressure self-monitoring, and frequent systolic blood pressure tests, as well as body weight and serum cholesterol values, are included in the instructional support. Food control, exercise therapy, hypertension problems, and their management will also be explored as non-pharmacological therapeutic approaches. Furthermore, based on their unique needs, patients will be informed about medicine administration timings and quantities. Finally, all patients will be informed about antihypertensive medications, including indications, side effects, contraindications, warnings/precautions, drug interactions, and pregnancy risks. Educational components will be delivered through educational booklets with animated infographics.

#### Involvement of patients and their families

A patient companion will be enrolled in the study for the duration of the trial and will participate in educational and counseling sessions. The objective of involving family members is to learn about their experiences with our Ed-counselling sessions.

#### mHealth intervention

The mHealth intervention will consist of daily written reminder messages and a video once a week. A professional Information and Technology (IT) facilitator will provide this intervention module. The IT facilitator will be responsible for overseeing the overall messaging, keeping track of it on the computer, and ensuring that all of the material for the various interventions is delivered on time. For the duration of the study, all participants will receive a free 12-month “WhatsApp” Internet connection.

A skilled IT team collaborated with two experts from one of the study hospital’s cardiology, hypertension, and nutrition departments in Lahore, Pakistan, to create educational animated infographic videos having both educational and counseling content. The content of the video is also based on the Health Beliefs Model. There are three parts to the video: (1) hypertension awareness, (2) uncontrollable hypertension’s consequences, and (3) improved health through medical and lifestyle improvements. The length of the video will be of 1 min.

Pilot testing among 10% of the total sample will be undertaken to guarantee that 165 people with hypertension and angina understood the video’s substance and message. These participants will be recruited from two of the study hospitals using the same criteria as indicated above. The results of this sample will not be included in the final analysis. As a result, these can be changed before the study. This will enable testing of the intervention and can establish if any errors occurred during the intervention’s delivery. It will also assist in identifying issues with eligibility requirements and determining the feasibility of obtaining a sufficient sample size.

Peer counseling and mobile health interventions are anticipated to help intervention group members adhere to their drug regimens and achieve the desired results. There are no limitations on concomitant care during the research.

### Follow-up

Subjects in all four arms of the study will be followed up, three times in 12 months following recruitment. Blood pressure measurement for treatment outcome will be done. The Self-efficacy for Appropriate Medication Adherence Scale (SEAMS) is an adherence-monitoring scale that will be used to collect information from participants regarding their adherence to medication self-reporting. Apart from SEAMS, self-reporting pill counting for measuring medication adherence will be used, and health-related quality of life using EuroQol 5-Dimension-3-level (EQ-5D-3L) and a visual analog scale (EQ-VAS) will also be assessed. All the above assessments will be done at baseline, 6 months, and 12 months of follow-up.

### Outcome measures

The primary outcome measure is the change in participants’ systolic blood pressure at 0, 6, and 12 months. It will be recorded by a nurse who will be oblivious to the study participants’ group. MODEL-605P YAMASU, a calibrated upper-arm mercury sphygmomanometer, will be utilized for this purpose. The blood pressure of the participant will be measured according to standard procedures. Two different readings will be collected within 5 min; the average measurement will be the final measurement. If the difference between the two measures is greater than 5 mmHg, a third evaluation will be performed, with the average of the two closest measurements being used as the final measurement [30].

In accordance with this, we are going to measure the proportion of participants who will achieve systolic blood pressure ≤ 140 mmHg. There are several secondary outcomes to be investigated. Health-related quality of life baseline, 6 months, and 12 months after the intervention will be measured via the EuroQol 5-Dimension- 3-level (EQ-5D-3L) [31, 32]. The five dimensions include mobility, self-care, normal activities, pain or discomfort, and anxiety or depression. Each dimension has three levels: no difficulty, moderate problems, and serious problems. In addition, we are using the EQ-VAS to measure the patient’s self-rated health on a vertical visual analog scale, with two unique endpoints such as “Best imaginable health condition” for a score of 100 and “Worst imaginable health state” for a score of 0 [31].

We will assess self-reported information regarding the number of tablets that will be utilized by participants within the specified timeline divided by the number of pills prescribed for that time multiplied by a hundred for the last 7 days. A rate of ≥ 80% will be considered adherent and vice versa [7, 8]; thereby, the percentage of participants who are successful in achieving adherent status between baseline and 12 months will be evaluated. In addition, to measure the adherence score at baseline, 6 months, and 12 months post-intervention, an Urdu/English version of the “Self-efficacy for Appropriate Medication Adherence Scale” (SEAMS) will be employed [33, 34]. The SEAMS is a 13-item measure for evaluating medication self-efficacy in chronic disease management that looks to be appropriate for people with low literacy abilities [34]. This survey will use a three-point response system, with 1 indicating lack of confidence, 2 indicating moderate confidence, and 3 indicating extreme confidence. The 13-item scale had a range of possible scores from 13 to 39. High scores indicate better adherence.

### Statistical analysis

The intention-to-treat analytic method will be conducted [35, 36]. Mixed-effects modeling with repeated measures will be employed for the primary outcome change in systolic blood pressure at 0, 6, and 12 months. To estimate treatment effects at each time point, an interaction between time and the groups will be fitted. For the primary and secondary outcomes measuring systolic blood pressure and medication adherence difference between and with the group at 0, 6, and 12 months, parametric (ANOVA/repeated measure ANOVA) and non-parametric tests *(*Kruskal*-*Wallis test*/*Friedman test/Wilcoxon signed*-*rank test) will be used. We shall account for the multiple time points statistically by using Tukey’s exact test. The Tukey test will be utilized for multiple comparisons and adjustment for multiple comparisons will be performed by Bonferroni correction. All the outcomes will be analyzed at 0, 6, and 12 months; however, the final analysis will be at 12 months from baseline. The *p*-value for the significance test will be set at *p* < 0.05. Pill counting data, adherence assessment at dichotomous variables, frequencies, and the chi-square test will all be used. By using the general estimating equation (GEE) with negative binomial regression, at 12 months post-follow-up, the covariates affecting primary and secondary outcomes including, age, gender, ethnicity, living status, education, monthly income, use of reminder, health insurance coverage, body mass index (BMI), diabetes, duration of illness, number of comorbid conditions, number of daily medication use, dose frequency will be determined and controlled. A complete analysis plan will be established for data analysis, including subgroup analyses and sensitivity analyses.

Sub-group analyses of the change in systolic blood pressure, the adherence to medication over the previous 12 months will be conducted by age (21–29 years, 30–49 years, 50 years and above), sex (male, female), adherence (SEAMS, pill-counting), and health-related quality of life (using EQ-5D-3L and EQ-VAS). Missing data from attrition will be handled using a multiple imputations approach at 12 months.

### Interim analysis

We will conduct an interim analysis at 6 months. Testing the measurements, questionnaires, response rate, and other study materials are the main objectives of this analysis. It will be used to evaluate the attrition rate, trends, and any difficulties or negative outcomes associated with the administration of the intervention. This analysis would not be reported as a result. The 12-month analysis is the final one.

### Data management

Two trained research assistants will gather data under the supervision of a research specialist. In sealed envelopes, all collected data will be delivered to a bio-statistician who will not be involved in any of the research activities and will be unaware of each participant’s assignment. All data must be entered twice, according to institutional regulation. The bio-statistician will clean and analyze the data. Stata version 16 (Stata corp SE) and R (version 4.0.3) will be utilized for all kinds of analysis in this study.

The research staff involved in data collection will include two research assistants and one research specialist. Two research assistants, in partnership with local hospital staff and under the supervision of a research specialist, will collect the data. A 2-day on-site training of research assistants on hypertension and its control, treatment, and how to respond to common questions about blood pressure and medication adherence including medication side effects will be conducted using a standard training module developed by a team of specialists from one of the study hospitals. They will also provide counseling and support to participants in filling out surveys. Data collection and randomized controlled trial procedure guidelines will be covered in three training sessions.

Each participant will receive a 5-min personal consultation to assist them in filling out the questionnaire, measuring their blood pressure, and counting their pills. Systolic blood pressure will be recorded by a nurse who will be oblivious to the study participants’ group. MODEL-605P YAMASU, a calibrated upper-arm mercury sphygmomanometer, will be utilized for this purpose. The blood pressure of the participant will be measured according to conventional procedures. Within 5 min, two different readings will be collected, with the average measurement being the final measurement. If the difference between the two measures is greater than 5 mmHg, a third evaluation will be performed, with the average of the two closest measurements being used as the final measurement.

Each of the participants will be given a paper-based questionnaire. Interviewers will read the questions to the participants and note down their answers if they have difficulty filling out the response. The paper-based questionnaires “Self-efficacy for Appropriate Medication Adherence Scale” (SEAMS) [34] contain socio-demographic, health-related, and adherence-related validated questions that would be employed in Urdu and English. The SEAMS is a 13-item measure for evaluating medication self-efficacy in chronic disease management that looks to be appropriate for people with low literacy abilities [34]. This survey used a three-point response system, with 1 indicating lack of confidence, 2 indicating moderate confidence, and 3 indicating extreme confidence. The 13-item scale had a range of possible scores from 13 to 39. In terms of drug adherence, greater ratings indicated higher levels of self-efficacy and vice versa. It is a dependable and precise device. Principal component factor analysis was used to determine the study’s validity. The researchers established a two-factor solution that accounted for 52.3% of the variance. Test-retest reliability had a moderate level of dependability (Spearman’s *r* = 0.62, *p* = 0.001). Internal consistency (Cronbach’s alpha = 0.89) is good [34].

The participants will be asked how many tablets they were prescribed for the period in question, as well as how many pills they took and missed during that time. Adherence rates will be calculated by dividing the number of pills ingested during a period by the number of tablets suggested for that period. Based on past research, an 80% cut-off value has been developed. Non-adherents will account for less than 80% of the rate, while adherents will account for more than 80% [7].

At the end of the sixth and twelfth months, participants who completed the baseline survey in all four groups will be invited to one of the study institutes to be questioned by qualified interviewers or over the phone who will be unable to come to complete the second round of blood pressure measurement, SEAMS filling, health-related quality of life post-intervention using EQ-5D-3L and EQ-VAS, and pill-counting information (Table 2).

**Table 2:**
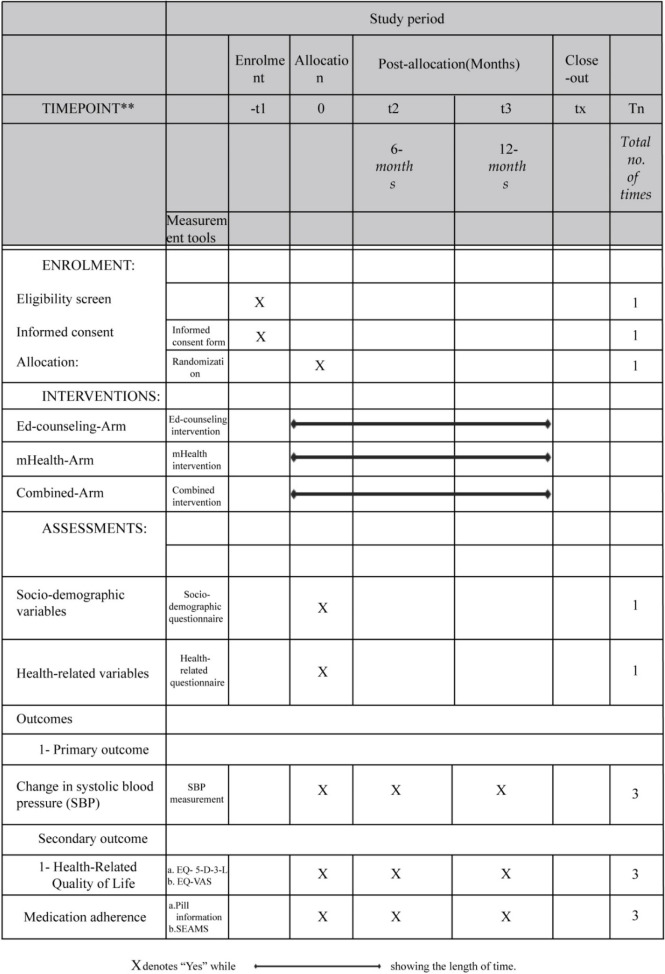
Content for the schedule of enrolment, interventions, and assessments

There will not be a data monitoring committee for this trial. This study does not necessitate a data monitoring committee but instead, we have an epidemiologist, biostatistician, and clinician to assess study integrity and its design aspects during the course of the study.

Representatives of the sponsor, coordinator, and ethical review board will visit the study locations and conduct audits, including the verification of source data. Additionally, there will be three monitoring visits each.

### Dissemination and access

After the trial is completed, the study’s final findings will be published in a peer-reviewed journal and shared with participants, healthcare providers, and various research organizations via a public access database. The results will be posted on clinicaltrials.gov after it is completed.

The data will be kept highly confidential. None of the research staff (including research assistants, investigators, and clinicians) will have access to the data except the principal investigator and the members of the data monitoring committee.

### Reporting of adverse events

Reporting of significant adverse incidents that might plausibly happen as a result of the trial will not be included as primary or secondary outcomes. All adverse incidents that are voluntarily reported by participants, the study team, or medical professionals will be documented [35].

### Communication of protocol amendments to relevant parties

Significant changes will be documented in process assessment and results publications and will be notified to the ethical committee and the ethical review boards of all study hospitals.

### Criteria for discontinuing or modifying interventions

Participants in the intervention groups will be contacted by phone by a member of our research team 1 month after the commencement of the intervention, and they will be interviewed. The interview will emphasize the participants’ satisfaction and comfort with the intervention. The study team will then be able to adjust the timing, duration, and frequency of some of the interventions or make further plans for adjustments.

### Ethical considerations

The study protocols were reviewed and approved by the Institutional Ethical Review Board, University of Punjab (No. IERB/ISCS/674). Additionally, the research protocol has been authorized and approved by the Rehmatul-Lil-Almeen Institute of Cardiology, Employers Social Security teaching hospital in Lahore (Reference number: RAIC-PESSI-1156). The study methods follow the Declaration of Helsinki and Good Clinical Practice, and the trial is registered with ClinicalTrials.gov (NCT05106790).

Trained research assistants will offer informed consent to participants who are voluntarily participating in the study. Confidentiality and privacy will be strictly followed. Participants’ privacy will be protected in a variety of ways: Any personnel participating will be required to sign an agreement guaranteeing the privacy of all information obtained. In addition, interviewers will receive training on the most effective way to maintain confidentiality. No real names or any other identity markers will be used in the study. Members’ names on the patient overview structures will not be referred to. The assent structures of the members will be placed in a secured cabin. Electronic data will be kept private and secure, and only the principal investigator will have access to it. No results will be directly related to the respondents [36].

The study is set up in such a way that the participants and medical workers are in as little danger as feasible. Participants face a minor risk in the form of a few mild psychological discomforts when answering questions about sensitive topics such as their own and their household’s income and may feel embarrassed when answering a follow-up questionnaire about medicine adherence due to their poor adherence status. To avoid such discomfort, the responder will be interviewed in a different room or at a place away from any witnesses. No post-trial compensation or care will be provided.

## Discussion

In LMICs, health intervention via mobile technology has the potential to reduce hypertension-related morbidity and mortality. The extensive use of mobile phones in LMICs like Pakistan may be a technique to improve treatment adherence. The purpose of this study is to see how effective an instructional assistance and reminder module as a mHealth technology intervention is at decreasing hypertension, patients” medication adherence, and health-related quality of life. It will also determine whether traditional mHealth methods, such as written messages and voice reminders, are more effective than multimedia pictures or videos, which is a whole new development in LMICs.

To our knowledge, this is the first study in Pakistan to create, compare, and evaluate the effectiveness of several mHealth intervention modules together. This intervention combines clinical principles and suggestions, as well as technological application ideas from the Health Belief Model. If this study shows significant effects in lowering the patient’s blood pressure and addressing the restrictions, it could be applied on a larger scale as a realistic strategy for increasing medication adherence in community settings.

In reality, this study addresses the challenging issue of the increasingly high prevalence of uncontrolled hypertension, limited adherence to long-term therapy, and their interlink. A step forward provides an assessment to adopt an effective and cost-effective way to overcome this obstacle which is acceptable in terms of the moral, cost, and social context of a community.

## Trial status

The study protocol has been registered at ClinicalTrials.gov (NCT05106790) on October 24, 2021. The recruitment started in December 2022 and will be finished by April 2023. The trial’s results are anticipated until May 2024.

## Supplementary Information

**Additional file 1.**

## Data Availability

After the trial is completed, the study’s final findings will be published in a peer-reviewed journal and shared with participants, healthcare providers, and various research organizations via a public access database. The results will be posted on clinicaltrials.gov after it is completed. On an appropriate request, the corresponding author can provide the protocol and statistical codes. Although the data will not be available publicly due to privacy.

## Abbreviations

IT: Information and Technology
LMIC: Low-and middle-income country
OPD: Outpatient Department
SEAMS: Self-efficacy for Appropriate Medication Adherence Scale
VAS: Visual analog scale

## References

[1] Mills KT, Stefanescu A, He J (2020). The Global epidemiology of hypertension. Nat Rev Nephrol.

[2] Kintscher U (2013). The burden of hypertension. EuroIntervention.

[3] NCD-Risk Factor Collaboration (2017). Worldwide trends in blood pressure from 1975 to 2015: a pooled analysis of 1479 population-based measurement studies with 19.1 million participants. Lancet.

[4] Lewington S, Clarke R, Qizilbash N, Peto R, Collins R (2002). Age-specific relevance of usual blood pressure to vascular mortality: a meta-analysis of individual data for one million adults in 61 prospective studies. Lancet.

[5] Bundy JD, Li C, Stuchlik P, Bu X, Kelly TN, Mills KT, He H, Chen J, Whelton PK, He J (2017). Systolic blood pressure reduction and risk of cardiovascular disease and mortality. JAMA Cardiol.

[6] National Institute of Population Studies (2019). Pakistan Demographic and Health Survey 2017–18.

[7] Osterberg L, Blaschke T (2005). Adherence to medication. N Engl J Med.

[8] Hashmi SK, Afridi MB, Abbas K, Sajwani RA, Saleheen D, Frossard PM, Ishaq M, Ambreen A, Ahmad U (2007). Factors associated with adherence to anti-hypertensive treatment in Pakistan. PloS One.

[9] Mahmood S, Jalal Z, Hadi MA, Orooj H, Shah KU (2020). Non-adherence to prescribed antihypertensives in primary, secondary and tertiary healthcare settings in Islamabad, Pakistan: a cross-sectional study. Patient Prefer Adherence.

[10] Jafar TH, Gandhi M, Jehan I, Naheed A, de Silva HA, Shahab H, Alam D, Luke N, Lim Wee (2018). Determinants of uncontrolled hypertension in rural communities in South Asia – Bangladesh, Pakistan, and Sri Lanka. Am J Hypertens.

[11] World Health Organization (2011). New Horizons for Health through Mobile Technologies. mHealth: new horizons for health through mobile technologies.

[12] Kamal AK, Shaikh Q, Pasha O, Azam I, Islam M, Memon AA, Rehman H, Akram MA, Affan M, Nazir S (2015). A randomized controlled behavioral intervention trial to improve medication adherence in adult stroke patients with prescription tailored short messaging service (SMS)-SMS4Stroke Study. BMC Neurol.

[13] Ni Z, Liu C, Wu B, Yang Q, Douglas C, Shaw RJ (2018). An MHealth Intervention to improve medication adherence among patients with coronary heart disease in China: development of an intervention. Int J Nursi Sci.

[14] Zhai P, Hayat K, Ji W, Li Q, Shi L, Atif N, Xu S, Li P, Du Q, Fang Y (2020). Efficacy of text messaging and personal consultation by pharmacy students among adults with hypertension: randomized controlled trial. JMIR.

[15] Chi BH, Stringer JSA (2010). Mobile phones to improve HIV treatment adherence. Lancet.

[16] Mawani M, Kadir MM (2016). Use of M-Health technology for preventive medicine in Pakistan. Curr Rev.

[17] Chatha ZF, Rashid U, Olsen S, ud Din F, Khan A, Nawaz K, Gan SH, Khan GM (2020). Pharmacist-led counselling intervention to improve antiretroviral drug adherence in Pakistan: a randomized controlled trial. BMC Infect Dis.

[18] M’imunya JM, Kredo T, Volmink J (2012). Patient education and counselling for promoting adherence to treatment for tuberculosis. Cochrane Database Syst Rev.

[19] Musayón-Oblitas Y, Cárcamo C, Gimbel S (2019). Counseling for improving adherence to antiretroviral treatment: a systematic review. Aids Care.

[20] Zaric GS, Bayoumi AM, Brandeau ML, Owens DK (2008). The cost effectiveness of counseling strategies to improve adherence to highly active antiretroviral therapy (HAART) among men who have sex with men. Med Decis Mak.

[21] Pakistan Bureau of Statistics. Available online: http://www.pbs.gov.pk/ (Accessed 11 April 2020).

[22] Chan A-W, Tetzlaff JM, Altman DG, Laupacis A, Gøtzsche PC, Krleža-Jerić K, Hróbjartsson A, Mann H, Dickersin K, Berlin JA (2013). SPIRIT 2013 Statement: defining standard protocol items for clinical trials. Ann Intern Med.

[23] Montgomery AA, Peters TJ, Little P (2003). Design, analysis and presentation of factorial randomised controlled trials. BMC Med Res Methodol.

[24] Whelan DB, Dainty K, Chahal J (2012). Efficient designs: factorial randomized trials. JBJS.

[25] Collins R, Peto R, MacMahon S, Hebert P, Fiebach NH, Eberlein KA, Godwin J, Qizilbash N, Taylor JO, Hennekens CH (1990). Blood pressure, stroke, and coronary heart disease. Part 2, Short-Term Reductions in Blood Pressure: Overview of Randomised Drug Trials in Their Epidemiological Context. Lancet.

[26] Lim C-Y, In J (2019). Randomization in clinical studies. Korean J Anesthesiol.

[27] Gallis JA, Bennett GG, Steinberg DM, Askew S, Turner EL (2018). Randomization procedures for multicomponent behavioral intervention factorial trials in the multiphase optimization strategy framework: challenges and recommendations. Transl Behav Med.

[28] Altman DG, Schulz KF (2001). Concealing treatment allocation in randomised trials. BMJ.

[29] Karanicolas PJ, Farrokhyar F, Bhandari M (2010). Blinding: who, what, when, why, how?. Can J Surg.

[30] Muntner P, Shimbo D, Carey RM, Charleston JB, Gaillard T, Misra S, Myers MG, Ogedegbe G, Schwartz JE, Townsend RR (2019). Measurement of blood pressure in humans: a scientific statement from the American Heart Association. Hypertension.

[31] EuroQol Group (1990). EuroQol – a new facility for the measurement of health-related quality of life. Health Policy.

[32] Rabin R, de Charro F (2001). EQ-5D: a measure of health status from the EuroQol GROUP. Ann Med.

[33] Culig J, Leppée M (2014). From Morisky to Hill-bone; self-reports scales for measuring adherence to medication. Coll Antropol.

[34] Risser J, Jacobson TA, Kripalani S (2007). Development and psychometric evaluation of the Self-efficacy for Appropriate Medication Use Scale (SEAMS) in low-literacy patients with chronic disease. J Nurs Measurement.

[35] Lewis JA, Machin D (1993). Intention to treat – who should use ITT?. Br J Cancer.

[36] Strobl J, Cave E, Walley T (2000). Data protection legislation: interpretation and barriers to research. BMJ.

